# Determination of Polycyclic Aromatic Hydrocarbons in Traditional Chinese Medicine Raw Material, Extracts, and Health Food Products

**DOI:** 10.3390/molecules27061809

**Published:** 2022-03-10

**Authors:** Chenggang Cai, Guoli Chang, Miaomiao Zhao, Pinggu Wu, Zhengyan Hu, Dingguo Jiang

**Affiliations:** 1 School of Biological and Chemical Engineering, Zhejiang University of Science and Technology, Hangzhou 310023, China; ccg0516@zust.edu.cn (C.C.); 212003817003@zust.edu.cn (G.C.); 212003817038@zust.edu.cn (M.Z.); 2 Zhejiang Province Center for Disease Control and Prevention, Hangzhou 310051, China; zhyhu@cdc.zj.cn; 3 China National Center for Food Safety Risk Assessment, Beijing 100021, China

**Keywords:** Chinese medicine raw materials, benzo(a)pyrene, four polycyclic aromatic hydrocarbon markers, herbal medicine

## Abstract

The four polycyclic aromatic hydrocarbon markers (PAH4) of benzo[a]anthracene (BaA), chrysene (Chr), benzo[b]fluoranthene (BbF), and benzo[a]pyrene (BaP) are indicators showing polycyclic aromatic hydrocarbon (PAH) contamination levels in Chinese medicine raw materials (CMRMs), extracts and health food products; Samples of herbal medicine, herbal extracts, and food supplements were extracted with *n*-hexane, then cleaned up sequentially on Florisil and EUPAH solid-phase extraction (SPE) columns. A gas chromatography–mass spectrometry method for the determination of four polycyclic aromatic hydrocarbon markers in Chinese medicine raw material, extracts, and health food products was established; In spiked-recovery experiments, the average recovery was about 78.6–107.6% with a precision of 2.3–10.5%. The limit of quantification (LOQ) and limit of detection (LOD) of the PAH4 markers in this method were 2.0 μg/kg and 0.7 μg/kg, respectively. When the developed method was utilized to determine PAH4 contents in 12 locally available health food products, 3 samples contained over 10.0 μg/kg BaP, and 5 samples contained over 50.0 μg/kg PAH4. The European Union (EU) limits for BaP and PAH4 are 10 and 50.0 μg/kg, respectively; therefore, more attention must be drawn to the exposure risk of BaP and PAH4 in CMRMs, their extracts, and health food products. According to the risk assessment based on the Margin of Exposure (MOE) method, it is recognized that the products mentioned in this study pose a low risk.

## 1. Introduction

Polycyclic aromatic hydrocarbons (PAHs) constitute a large class of organic compounds that contain two or more fused aromatic rings composed of carbon and hydrogen atoms [1]. PAHs are given priority concern because of their mutagenic and carcinogenic effects [2]. PAHs can be generated during the food preparation process, such as baking, broiling, frying, grilling, or smoking [3,4,5,6,7,8]. Many foods have been reported to contain PAHs: tea [1,9,10], coffee [10], and meat [11], and there are many other foods or food materials containing PAHs that have not been of concern in the traditional consumption habits or utilization methods. For example, some plant foods or ingredients involving herbs or their extracts were rarely reported for their pollution or contamination by PAHs. Due to the increase in trade, including food and some traditional herb medicine worldwide, some regulations were established for the control of PAHs import or export. For example, the European Commission (EC) has enacted EU Regulation 2015/1933 [12], which defines the maximum allowable residual content of benzo[a]pyrene (BaP) and PAH4 of benzo[a]anthracene (BaA), chrysene (Chr), benzo[b]fluoranthene (BbF) and BaP in food supplements, dried herbs, and dry spices as a representative assessment of overall PAHs risk. The BaP content in dry herbs should be less than 10.0 μg/kg, and the total PAH4 content should be less than 50.0 μg/kg.

The Chinese medicine raw materials (CMRMs) are the raw material of Chinese medicinal plants and herbs; the materials can be used as medicine after cooking or prepared to product by factory. The utilization of CMRMs, usually plants and herbs or their extracts, in everyday food is common in China, especially those CMRMs mentioned in the article “One Root of Medicine and Food” [13]. To date, there are more than 100 CMRMs documented by the National Health Commission of the People’s Republic of China. Most of the CMRMs are prepared from traditional herbs; when they are grown in a field, the PAHs in the environment will contaminate the plant herbs, and PAHs in lubricating oil of the machines or other materials such as solvents will also contaminate the CMRMs in the processing procedures [14]. Apart from the external sources, some medicine materials need a long time cooking at high temperatures and may lead to the internal PAHs formation. For example, some medicines need frying pretreatment to produce better functional effects or roasting to form a special shape for traditional habits or convenient storage, which may cause the internal formation of PAHs. Currently, there are no official standardized determination methods and limit requirements for PAHs in CMRMs in China. The closest existing national standard, “Food Safety National Standard: Food Determination of Polycyclic Aromatic Hydrocarbons” in GB5009.265, utilizes high-performance liquid chromatography (HPLC)-fluorescence detector (FLD) and Gas Chromatography–Mass Spectrometry (GC–MS) methods for PAHs detection in food; however, CMRMs are excluded. Due to the complexity of CMRMs, the pigments found in these foods can influence the PAHs’ purification process. The existing liquid–liquid extraction methods and SPE column purification methods in GB5009.265 do not meet the purification effectiveness required for accurate PAHs quantification in our trial experiment. The purification effects using the SPE column will be further tested in this study. Other analysis methods for PAHs determination in CMRMs, using the HPLC/FLD [14,15] and GC–MS/MS [16], were also performed. For the extraction and purification steps, Gel Permeation Chromatography (GPC) and solid-phase extraction (SPE) purification were used for PAHs analysis in the *Rhizoma coptidis* products [16] while other reports analyzed PAHs after liquid extraction and concentration [14,15]. Considering the different kinds of CMRMs varying in their compositions, it is necessary to analyze other samples for the survey of PAHs contamination levels, and the purification method needs further study.

The SPE columns used for purification include Florisil, EUPAH SPE (styrene-divinylbenzene polymer), *n*-propyl ethylenediamine (PSA), SDB (styrene-divinylbenzene copolymer), silica, aminopropyl, C_18_ SPE columns, and others [8,9,11,17,18,19,20,21]. Usually, the columns alone were used for PAHs purification after liquid solvent extraction, but there are also combinations using GPC and SPE clean-up for cleaning impurities before analysis of vegetable oil samples [22], or other clean-up combinations of PRS (propylsulphonic acid) SPE and silica cartridge for beef meat [23], C_18_ and PSA for dry fermented sausage [24], animal-based smoked foods [25], salmon, shrimps and other samples [26]. According to the complexity of CMRMs compositions, it is necessary to establish a feasible method for purifying the PAHs in the samples. Until now, there have been no procedures for the purification of PAHs in the CMRMs related products, using two SPE columns. This article established a new method with a two-step SPE purification procedure for PAHs analysis, and the developed method was applied to determine PAHs in 12 representative CMRM-containing health food products. Finally, the method of exposure limits, Margin of Exposure (MOE), was utilized to assess the PAHs exposure risk in the tested samples.

## 2. Results and Discussion

### 2.1. Analysis Conditions

**Figure 1:**
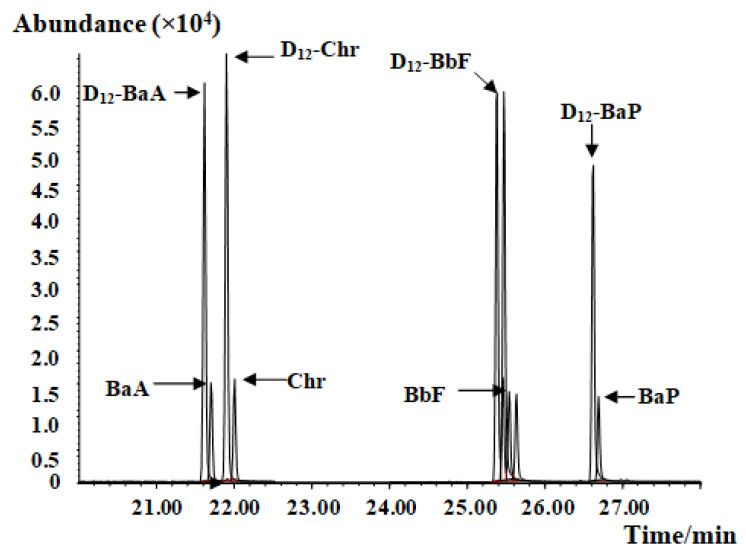
Mass chromatograph of the PAH4 and the D_12_-PAH internal standard.

GC–MS parameters and PAH analysis conditions utilized were those found in GB 5009.265 [27]. The mass chromatogram of PAH4 and their internal standards are shown in Figure 1. The standard working solution was determined, and the corresponding peak areas of PAH4 and internal standard D_12_-PAH4 were measured. In the measurement range of 0.005 µg/mL to 0.01 µg/mL, the concentration ratio of PAH4 to 0.05 µg/mL internal standard D_12_- PAH4 was set to the abscissa x, and the peak area ratio of PAH4 to internal standard D_12_-PAH4 (response ratio) was set to the ordinate y to obtain a linear Equation. The monitoring ion and linear Equation of each compound are shown in Table 1.

### 2.2. Extraction

The extraction of PAHs from traditional CMRMs, using direct organic solvent extraction, required cyclohexane, ethyl acetate, and mixed *n*-hexane and acetone systems; however, some samples were difficult to process and did not disperse finely in the extraction solvent, leading to incomplete extraction. To address this clumping issue, anhydrous ethanol and water were firstly used to wet the CMRMs. After samples are fully wetted and dispersed, organic solvents can effectively extract the PAHs from the sample. In this study, anhydrous ethanol and hexane were employed for sample wetting and sample extraction, respectively.

### 2.3. Purification

**Figure 2:**
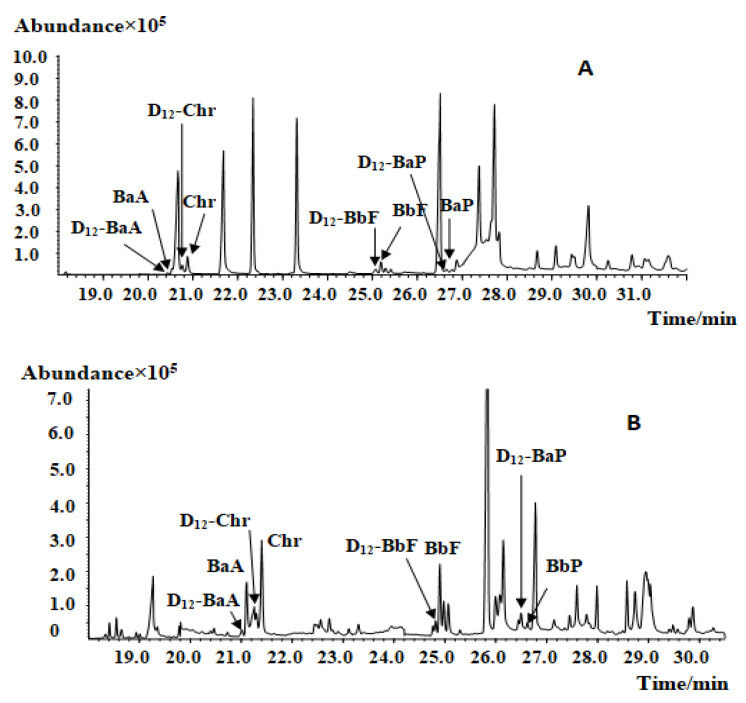

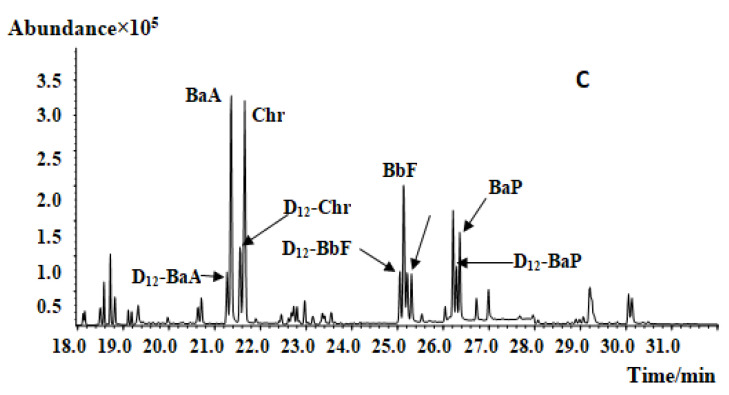
Mass chromatogram of Lotus leaf extract before and after SPE purification: (**A**) before purification; (**B**) after FLORISIL SPE purification; (**C**) after FLORISIL and EUPAH SPE columns purification.

Due to the various plant parts required in traditional Chinese medicines (roots, stems, leaves, flowers, and fruits), the extracts of CMRMs [15], especially ethanol extracts, can contain high amounts of pigments and volatiles, which will influence PAHs analysis; therefore, purification of the crude extract is very important for effective PAHs analysis. GPC can effectively remove selected pigments, fats, and other interfering substances, but small and low molecular weight compounds have low removal efficiency. Furthermore, GPC consumes large amounts of organic reagents, making it a costly and nonideal purification method. A saponification-based purification procedure can remove chlorophyll, fat, and selected other interfering components; however, saponification cannot remove carotene and volatile oil-rich samples. Instead of GPC or saponification, this study employed an SPE strategy to address the need for sample purification prior to GC–MS analysis. The purification effects of the Florisil SPE column and Florisil and EUPAH SPE columns were carried out (Figure 2). The result showed that, after purification, the impurities decreased, especially the peaks from 21 to 25 min and those after 28 min. Moreover, the peaks of PAH4 were separated after the two columns were used. All the samples were purified by the two SPE columns before analysis in this study.

In “National Food Safety Standard Determination of Polycyclic Aromatic Hydrocarbons in Foods”, a PSA column and a C_18_ SPE column were used for PAHs purification from grains and low-water food samples. For high fat or oil samples, a Florisil SPE column was selected. In this study, a 2 g/12 mL Florisil column was used because it can effectively adsorb chlorophyll, lutein, and some of the interfering oils. The EUPAH SPE column can specifically adsorb PAHs and remove the interference of alkane and acidic compounds, thus enabling quantitative analysis. For the pigment-rich samples, it was necessary to decrease the sample load to avoid overloading the SPE column.

### 2.4. Accuracy, Precision, and Quality Control of the Developed Method

**Figure 3:**
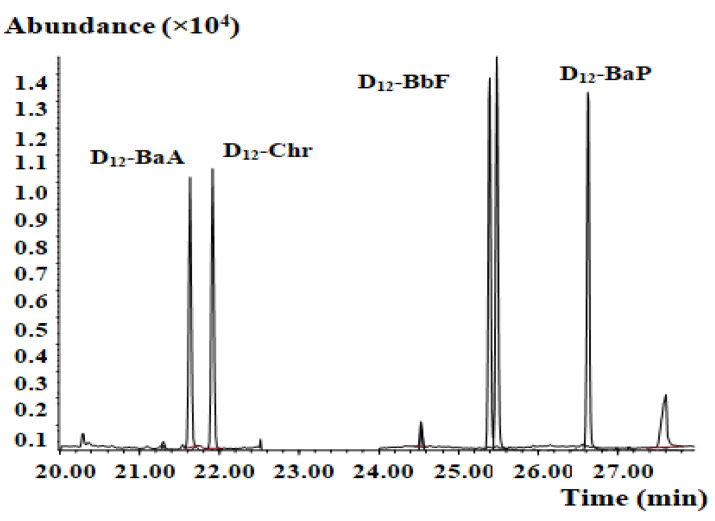
Mass chromatogram of the blank test.

PAHs are ubiquitous environmental pollutants, and external PAH contamination must be strictly controlled during the experimental process. Reagents, vessels, concentrators, and consumable laboratory supplies are the main sources of external contamination. To reduce the PAH4 blank background value, pollutants were removed from vessels using high-temperature baking at 300 °C or hexane washing. Reducing reagent consumption and the step-count can also effectively control the blank value. In this study, the blanks contained less PAH4 than the detection limit of the method, which effectively ensured the reliability of the results. The blank test mass chromatogram of the developed method is shown in Figure 3.

Three concentrations of PAH4 (2.0 μg/kg, 10.0 μg/kg, and 20.0 μg/kg) were spiked into three sample groupings: unprocessed *Dendrobium candidum*, *Dendrobium candidum* extract, and *Dendrobium candidum*-containing health food product (n = 6 for each group) for a recovery test. The accuracy and precision of the method are shown in Table 2. The average recovery was 78.6–107.6%, and the precision was 2.3–0.5% when 2.0 μg/kg, 10.0 μg/kg, and 20.0 μg/kg of PAH4 were spiked in unprocessed *Dendrobium candidum*, *Dendrobium candidum* extract, and *Dendrobium candidum*-containing health food product, respectively. These results meet the criteria specified in EU regulation recovery between 50% and 120%. Therefore, when a 1.00 g sample is selected, the LOQ and LOD of the four PAHs compounds are 2.0 μg/kg and 0.7 μg/kg, respectively.

### 2.5. Samples Analysis

**Figure 4:**
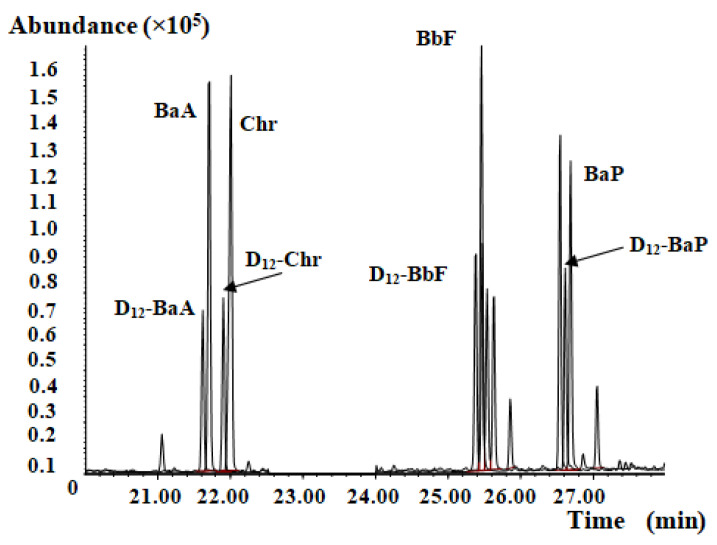
Representative mass chromatogram for a traditional Chinese medicine extract.

**Figure 5:**
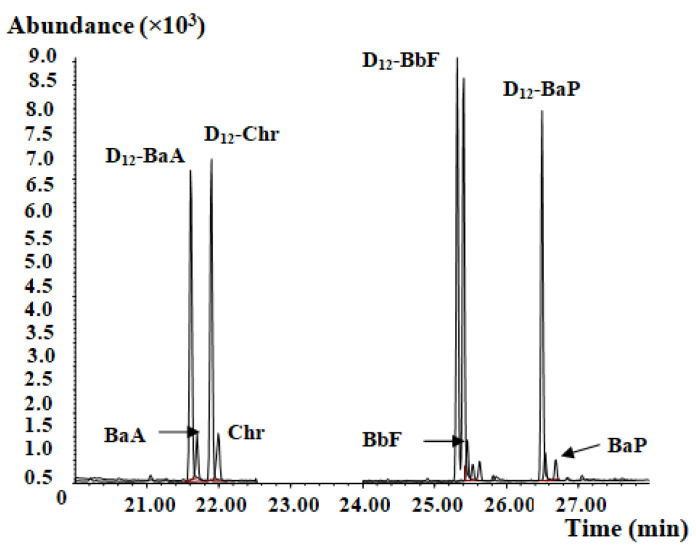
Representative mass chromatogram of CMRM-containing health food.

CMRM extracts are much more commonly employed than unprocessed CMRM for health food product production. The PAH4 content for 12 samples of traditional Chinese medicine extracts and health foods containing CMRMs was analyzed using the developed method three times. The results are presented in Table 3. The BaP content in 3 of the 12 sampled materials exceeded the acceptable EU regulation No. 2015/1933 tolerance limit of 10.0 μg/kg. According to the same regulation, the total PAH4 content tolerance limit of 50.0 μg/kg was exceeded by 5 of the 12 sampled materials. The test results in Table 3 showed that the total PAH4 is more likely to exceed the EU acceptable limits, and this requirement would be more difficult to control than limiting the levels of a single PAH species. Representative chromatograms of PAH4 analysis for CMRM-containing extracts or health food are presented in Figure 4 and Figure 5. In addition, the developed method was verified for effectiveness by analysis of PAH4 in *Rhodiola rosea* extract and spirulina. The results showed that the spirulina contained more BaP and PH4 than the *Rhodiola rosea* extract (Table 4).

The current status of PAHs pollution in Chinese herbal medicines had been previously studied; the methods were different from the sample preparation and analyzed methods. A total of 16 PAHs were determined in 79 Chinese herbal medicines by GC–MS/MS; the total PAHs concentrations were in the range of 21.1–2856.0 µg/kg, for different sample types, and the PAHs levels of flowers, leaves, roots, and stems were higher than those of fruits and seeds [15]. Four samples in eight *Coptis chinensis* Franch products contained BaP below 5 μg/kg, and the samples were analyzed by HPLC/FLD [16]. BaPs ranging from 1.67 to 94.9 μg/kg found in the marigold extract were detected by HPLC/FLD [14]. Ten kinds of *Red clover* extract, *Hypericum perforatum* extract, *Valeriana officinalis* extract as well as *Giant knotweed* extract were surveyed for PAH4 concentration by GC–MS, combined with sulfuric acid acidification, and the results showed PAH4 content ranging from 19.59 to 142.69 μg/kg [28]. PAH4 in 68 samples (total 94 health foods) exceeded the level for PAH4 compounds [29]. Although some of the reports concerned CMRMs and related products, they were relatively less analyzed compared with other foods for PAHs concentration. Because there are many kinds of CMRMs and related products, the results of this study will contribute more information on PAH levels in CMRMs products. Considering the popularity of CMRM-containing products, the reported presence of PAHs in these products will significantly increase the consumers’ daily exposure to PAHs, making it very important for ‘health foods’ to be monitored for PAH content. According to Table 3, the content of BaPs and PAH4 in CMRM extracts exceeded the EU limit requirement of 10 and 50 µg/kg, respectively; therefore, health food products containing these two raw materials have the potential to exceed the safe consumption limits.

PAHs are found in a variety of foods, from CMRMs and related products to edible oils, smoked, and barbecued foods [30,31,32]. Most of the CMRMs are traditional Chinese herbs cultivated in fields; thus, it is difficult to avoid the PAHs contamination from the air and water [33,34,35,36], and the processing procedure will influence the change of PAHs, especially by frying or roasting at high temperature for a long time. There are rarely any documents concerning the change of PAHs during CMRMs processing at high temperatures. The traditional extraction method will introduce the PAHs into the extraction system if the solvent is polluted by PAHs [14], while the concentration or dry steps after extraction will improve the PAHs concentration in the extracted product. Thus, the CMRMs in the tested sample contained a high level of PAHs (Table 3). As for the health food, the formulas are different in kinds and quantities of the CMRMs extract; the PAHs are relatively lower than in the pure extract (Table 3) because other ingredients such as sweeteners and dispersants were added into the formula.

Because PAH contamination is ubiquitous in the environment, traditional CMRMs can become polluted during cultivation or harvesting/processing [14]. Thus, it is important to pay more attention to the PAHs content in CMRM extracts and their resulting health foods. To decrease PAHs content and improve consumer health, standard acceptable tolerance limits must be established for products utilizing CMRMs.

### 2.6. Exposure Assessment

The MOE method is used to assess the risk characteristics for substances with genetic toxicity, which is published by the U.S. Environmental Protection Agency (U.S. EPA) and preferred by both the World Health Organization (WHO) and the European Food Safety Authority (EFSA) for the risk evaluation of carcinogens. Generally, it was assumed that a MOE of 10,000 or higher would be of low concern for public health and might be considered of low priority for risk management actions [37]. The benchmark dose level 10 (BMDL_10_), an estimate of the lowest dose (95% confidence level) that causes no more than a 10% cancer incidence in rodents, was used to obtain the MOE. Taking primary hepatocellular carcinoma as the toxicity effect endpoint to assess the risk of PAHs intake in the population, the BMDL_10_ of BaP and PAH4 were 70 µg/kg bw/d and 340 µg/kg bw/d, respectively.

Health food products are usually recommended for consumption at 3 g/day. According to Table 3, the highest measured content of PAH4 in the health food products category (Health Food 1, BaP and PAH4 of 76.3 μg/kg and 238.3 μg/kg, respectively), for the maximal daily exposure of BaP and PAH4 were of 228.9 and 714.9 ng/day. For an adult with the body weight (BW) calculated as 70 kg, the EDI of BaP and PAH4 was 3.27 ng/d BW and 10.21 ng/d BW, respectively. Based on the MOE calculation method, the highest values of BaP and PAH4 are 21,406 and 33,300, respectively. Since it was recognized that a MOE of 10,000 or higher would be of low concern for public health, the MOE values of BaP and PAH4 were of little concern. The result of BaP is less than the maximum estimated daily intake of BaP for a 70 kg person of 6–8 ng/kg [38]. Considering our consumption habit, and that those with compromised health will eat healthy foods, it is recognized that the products mentioned in this study pose a low risk.

## 3. Materials and Methods

### 3.1. Chemical Reagents and Samples

The 10.0 μg/mL PAH4 (BaA, Chr, BbF, and BaP) mixed standard solution (LA20950183CY PAH-Mix183) and the 100 μg/mL D_12_-PAH4 (D_12_-BaA, D_12_-Chr, D_12_-BbF, and D_12_-BaP) mixed internal standard solution (XA20950902CY PAH-Mix9) were purchased from Dr. Ehrenstorfer GmbH Corp (Augsburg, Germany).

Florisil (2 g/12 mL), EUPAH SPE (styrene-divinylbenzene polymer, 300 mg/6 mL), n-propyl ethylenediamine (PSA), and C_18_ SPE columns were purchased from Kang Yuan Technology Co., LTD. (Hangzhou, China). Acetone, isooctane, ethyl acetate, and *n*-hexane of chromatography grade were purchased from Merck, USA. Analytical grade anhydrous ethanol, anhydrous ether, petroleum ether, methylene chloride, concentrated aqueous ammonia, potassium hydroxide, and anhydrous sodium sulfate were purchased from Sinopharm (Shanghai, China). Ultrapure water was obtained using a Milli-Q water polishing system. To reduce the blank background value, vessels, glass tubes, and containers were baked at 300 °C or washed with hexane. Total of 12 representative CMRM extracts and health food samples, including 2 *Dendrobium candidum* extract (contain polysaccharides), 1 *Astragalus mongholicus* extract (contain flavonoids), 1 *Codonopsis pilosula* extract (contain glycosides), 1 *fructus alpiniae oxyphyllae* extract (contain essential oils), 1 *Gastrodia elata* extract (contain glycosides), 1 *Ganoderma lucidum* extract (contain terpenoids), 2 *Dendrobium candidum*-containing health foods, 2 *Ganoderma lucidum*-containing health foods, and 1 *Codonopsis pilosula*-containing health food as well as *Rhodiola rosea* extract, Lotus leaf extract, and spirulina were purchased from a local market in Hangzhou, China.

### 3.2. Analytical Instrumentation

An Agilent 6890–5973 gas chromatography–mass spectrometer (GC–MS), equipped with a DB-PAHEU capillary column (20 m × 0.18 mm inner diameter × 0.14 μm film thickness) part number 121–9627, was used for sample analysis. The injection port temperature was 280 °C.

The column temperature was initially set to 80 °C for 2 min, then increased to 250 °C in 10 °C/min intervals, maintained at 250 °C for 2 min, and finally set to 320 °C in 8 °C/min intervals and maintained at 320 °C for 5 min. High purity helium (99.999%) was used as the carrier gas. Splitless sample injection (1 μL) was employed, and the sample was ionized by an electron ionization source at 70 eV and 230 °C, with a 250 V electron multiplier and a 280 °C transmission line temperature. The monitoring ions are listed in Table 1.

Additional equipment was obtained from their respective manufacturers as follows: multisample vortex oscillator (Heidolph Corp. Ltd., Schwabach, Germany); traditional Chinese medicine ultrafine pulverizer (Hangzhou Xuzhong mechanical equipment co., Ltd., Hangzhou, China); high-speed centrifuge (Beckman, Brea, CA, USA); solid-phase extraction manifold (CNW Corp. Ltd., Qingdao, China), nitrogen blowing instrument (Anpu Corp. Ltd., Shanghai, China); analytical balance with a sensitivity of 0.01 g (Mettler, Toledo, Columbus, OH, USA); and ultrasonic cleaner (Kunshan ultrasonic instrument Corp., Ltd., Kunshan City, China).

### 3.3. Preparation of Standard Solutions

The 0.2 μg/mL PAH4 mixed standard stock solution was prepared by diluting 10.0 μg/mL PAH4 mixed standard solution (0.2 mL) with acetone (9.8 mL). The 0.2 μg/mL D_12_-PAH4 mixed internal standard stock solution was prepared by diluting 100.0 μg/mL D_12_-PAH4 mixed internal standard solution (0.02 mL) with acetone (10.0 mL). The D_12_-PAH4 stock solution was stored in the dark at 4 °C. Five working solutions containing 1.0, 2.0, 5.0, 10.0, or 20 ng PAH4 and 10 ng D_12_-PAH4 were prepared for the GC–MS analysis as internal standards by aliquoting 5 μL, 10 μL, 25 μL, 50 μL, or 100 μL of the 0.2 μg/mL PAH4 standard solution to five volumetric flasks already containing 50 μL of 0.2 μg/mL D_12_-PAH4 mixed standard solution; the final working solutions containing 0.0025, 0.005, 0.01, 0.025, 0.050 μg/mL PAH4 and 0.025 μg/mL D_12_-PAH4, respectively. All the stock solutions were stored in the dark at 4 °C.

### 3.4. Sample Preparation

#### 3.4.1. Sample Extraction

Solid CMRM samples were crushed using a superfine pulverizer and stored in 50 mL plastic centrifuge tubes until required. Chinese medicine extracts were directly subject to the extraction procedure without additional processing.

The PAH extraction procedure was as follows: 2.00 g of sample was placed in a 50 mL centrifuge tube, and 50 μL of 0.2 μg/mL D_12_-PAH4 mixed internal standard solution as well as 10 mL anhydrous ethanol were added. The mixture was subject to ultrasonic treatment for 5 min, and then 10 mL of water was added. The resulting mixture was vortexed for 1 min and ultrasonicated for another 5 min. Finally, 10 mL of *n*-hexane was slowly added, and the mixture was vortexed for an additional 5 min. After centrifugation at 9000× *g* for 3 min, the organic *n*-hexane phase was collected for further purification.

#### 3.4.2. Sample Purification

Before purification, the Lotus leaf extract without purification, purified by the Florisil column and by the Florisil and EUPAH SPE columns, were analyzed for comparison of the purification method.

The SPE purification procedure [25] was as follows: anhydrous sodium sulfate (1 g) was added to a Florisil column, and *n*-hexane (5 mL) was passed through to activate the SPE column. The *n*-hexane sample extract about 10 mL collected previously was directly loaded on the activated column, and the eluent was collected in a 15 mL centrifuge tube for PAHs collection. Then, in order to fully collect PAHs, another 5 mL of methylene chloride *n*-hexane solution (3:17, *v:v*) was eluted through the column, and the eluent was collected in the same 15 mL tube. The collected combination liquid (about 15 mL) was concentrated under a stream of N_2_ at 50 °C and reconstituted in 2 mL *n*-hexane. The EUPAH SPE column was first activated with 3 mL methylene chloride and 3 mL *n*-hexane. Thereafter, the reconstituted 2 mL *n*-hexane sample was loaded to the column and eluted at 1.0 mL/min. *n*-Hexane (4 mL) was passed through to remove the impurities, then 5 mL of methylene chloride: ethyl acetate solution (1:1, *v:v*) was eluted through the column. The eluent was collected in a 10 mL glass tube and concentrated under a stream of N_2_ at 40 °C. The obtained residue was reconstituted in 200 μL acetone: isooctane (1:1, *v:v*) and transferred to a sample vial for GC–MS analysis. Blank test controls were conducted together with the samples for analyses.

### 3.5. Quality Control

Concentrations of PAH4 (2.0 μg/kg, 10.0 μg/kg, and 20.0 μg/kg) were spiked into unprocessed *Dendrobium candidum*, *Dendrobium candidum* extract, and *Dendrobium candidum*-containing health food products (n = 6 for each group) for a recovery test. The limit of detection (LOD) at a signal-to-noise ratio (S/N) of 3 and the limit of quantification (LOQ) at an S/N of 10 were determined from the S/N ratio check function on low PAH-containing samples in MSD ChemStation software (Agilent Technologies).

### 3.6. Data Analysis

The experimental data are represented as mean ± standard deviation (SD) of three independent replicates. The calculation of SD and linear equations were conducted with the SPSS 20.0 software package (SPSS Inc., Chicago, IL, USA).

### 3.7. Risk Assessment for Dietary Exposure to PAHs

The MOE value is the ratio between the reference dose calculated by the dose–response relation curve and the human dietary exposure estimated by the mathematical model. In order to estimate MOE, the chronic daily intake (CDI) of PAHs using the BaP equivalent concentration should be calculated according to the Environmental Protection Agency (EPA) [39]. The ratio between benchmark dose lower confidence limit (BMDL) and chronic daily intake (CDI) can be considered as MOE; the CDI value can be calculated by the estimated daily intake (EDI) value U.S. EPA’s benchmark dose software [40].

The MOE calculation formula is as follows:

MOE = BMDL_10_/EDI (1)

where MOE is the margin of exposure for BaP or PAH4 (dimensionless), BMDL_10_ is Benchmark Dose Level 10, which means the lowest dose that causes adverse reactions in 10% of the population, the BMDL_10_ value of BaP and PAH4 were of 70 µg/kg bw/d, and 340 µg/kg bw/d, respectively [41].

## 4. Conclusions

An isotope internal standard dilution combined with a GC–MS detection method was developed to determine the content of PAH4 in CMRM-containing extracts and health food products. CMRM samples were extracted with *n*-hexane and sequentially cleaned up by SPE on Florisil and then a EUPAH SPE column.

In spiked samples containing 2.0 μg/kg, 10.0 μg/kg, or 20.0 μg/kg PAH4, the average recovery was 78.6–107.6% with a precision of 2.3–10.5%, which can meet the detection requirement limits for BaP (10.0 μg/kg) and PAH4 (50.0 μg/kg) in herbal medicine, and food supplements containing botanicals according to EU regulation 2015/1933. The limit of quantification (LOQ) and limit of detection (LOD) of the PAH4 markers in this method were 2.0 μg/kg and 0.7 μg/kg, respectively. This method for PAH4 analysis was applied to 12 commercially available CMRM-containing food products. Using the tolerance limits established by EU regulation 2015/1933, three samples exceeded the tolerance limit for BaP content, and five samples exceeded the tolerance limit for total PAH4 content. Finally, the method of exposure limits was utilized to assess the PAHs exposure risk in the tested samples; it is recognized that the products mentioned in this study are of low risk. To encourage the development of better processing methods in CMRM-containing health product production that can remove PAHs and decrease PAH contamination, regulations and limits for safe levels of PAH in CMRM-containing products should be enacted.

## Figures and Tables

**Table 1:** Chemical monitoring ions and linear equations.

Chemicals	Identification Ions	Internal Standards	Identification Ions ^1^	Linear Equations	R^2^	Limit Range (×10^−^^3^ µg/mL)
BaA	226, 229, 228 ^1^	D_12_-BaA	236, 240	y = 0.418x − 0.022	1.0	2.5–50
Chr	226, 229, 228 ^1^	D_12_-Chr	236, 240	y = 0.352x − 0.018	0.999	2.5–50
BbF	250, 253, 252 ^1^	D_12_-BbF	260, 264	y = 0.296x + 0.011	0.999	2.5–50
BaP	250, 253, 252 ^1^	D_12_-BaP	260, 264	y = 0.283x + 0.017	0.999	2.5–50

^1^ are the quantitative ions.

**Table 2:** Recovery of PAH4 from *Dendrobium candidum*-containing samples (n = 6).

Samples	Standard Spiked (μg/kg)	BaA Average Recovery (%)	RSD%	Chr Average Recovery (%)	RSD%	BbF Average Recovery (%)	RSD%	BaP Average Recovery (%)	RSD%
Raw *Dendrobium candidum*	2.0	86.2	9.2	88.2	10.5	85.9	8.3	89.8	8.9
	10.0	92.8	7.6	94.2	8.5	96.2	7.3	103.4	6.2
	20.0	98.7	4.3	102.3	5.1	105.3	4.2	107.6	3.8
*Dendrobium candidum* extract	2.0	78.6	9.2	82.5	10.5	80.2	8.3	83.0	8.9
	10.0	81.0	7.6	88.9	8.5	86.0	7.3	92.6	6.2
	20.0	90.6	4.3	92.7	5.1	97.5	4.2	102.4	3.8
*Dendrobium candidum*-containing health food	2.0	81.2	8.3	86.4	7.4	80.5	7.8	81.6	8.4
	10.0	88.4	7.1	92.3	6.7	91.2	6.4	94.9	4.9
	20.0	98.1	3.2	99.4	3.4	98.6	2.9	101.4	2.3

**Table 3:** PAH4 content of 12 samples containing CMRM extracts (μg/kg).

Samples	BaA	Chr	BbF	BaP	PAH4
Extract 1	238.2 ± 11.9	214.2 ± 10.7	99.2 ± 4.96	92.9 ± 4.64	644.5 ± 32.2
Extract 2	19.2 ± 0.77	26.8 ± 1.07	3.2 ± 0.127	2.4 ± 0.1	51.6 ± 2.06
Extract 3	24.2 ± 1.45	10.2 ± 0.61	4.5 ± 0.27	3.6 ± 0.22	42.5 ± 2.55
Extract 4	12.8 ± 0.38	16.8 ± 0.5	8.1 ± 0.24	2.4 ± 0.07	40.1 ± 1.20
Extract 5	3.2 ± 0.23	5.7 ± 0.4	1.6 ± 0.11	1.3 ± 0.09	11.8 ± 0.83
Extract 6	12.7 ± 0.38	30.5 ± 0.92	6.4 ± 0.19	5.8 ± 0.17	55.4 ± 1.67
Extract 7	259.1 ± 5.19	553.8 ± 11.1	445.4 ± 8.92	755.0 ± 15.1	2013.3 ± 40.3
Health food 1	55.6 ± 3.34	65.8 ± 3.95	40.6 ± 2.43	76.3 ± 4.58	238.3 ± 14.3
Health food 2	5.1 ± 0.36	8.3 ± 0.58	5.8 ± 0.41	5.4 ± 0.38	24.5 ± 1.72
Health food 3	1.0 ± 0.08	1.8 ± 0.15	0.6 ± 0.05	ND ^1^	3.7 ± 0.31
Health food 4	3.9 ± 0.31	9.5 ± 0.76	2.4 ± 0.19	2.1 ± 0.17	17.9 ± 1.43
Health food 5	3.6 ± 0.26	16.2 ± 1.15	3.7 ± 0.26	0.8 ± 0.06	24.3 ± 1.7

^1^ Not Detected.

**Table 4:** Results of PAH4 in *Rhodiola Rosea* L. extract and spirulina.

Samples	Chemicals	Analysis Results (μg/kg)	Limit Range (μg/kg)
*Rhodiola Rosea* Extract	BaA	3.70	2.7 ± 1.2
	Chr	6.79	-
	BbF	2.36	1.7 ± 0.76
	BaP	1.66	1.2 ± 0.54
Spirulina	PAH4	14.5	-
	BaA	3.31	4.1 ± 1.8
	Chr	17.97	12.7 ± 5.6
	BbF	6.39	9.4 ± 4.2
	BaP	2.06	3.0 ± 1.32
	PAH4	29.72	29.0 ± 12.8

## Data Availability

All the data are included in this manuscript.

## Sample Availability

Samples of the *Dendrobium candidum* extract, *Astragalus mongholicus* extract, *Codonopsis pilosula* extract, *fructus alpiniae oxyphyllae* extract, *Gastrodia elata* extract, *Ganoderma lucidum* extract, *Dendrobium candidum*-containing health foods, *Ganoderma lucidum*-containing health foods, and *Codonopsis pilosula*-containing health food as well as *Rhodiola rosea* extract, Lotus leaf extract, and spirulina are available from the authors.
